# Women With Bipolar Disorder are at Increased Risk of Sarcopenia

**DOI:** 10.1111/bdi.70192

**Published:** 2026-10-19

**Authors:** Lana J. Williams, Amanda L. Stuart, Michael Berk, Shae E. Quirk, Emma C. West, Heli Koivumaa‐Honkanen, Risto Honkanen, Jeremi Heikkinen, Jessica A. Davis, D. Kavindi Weerasinghe, Julie A. Pasco

**Affiliations:** ^1^ Institute for Mental and Physical Health and Clinical Translation (IMPACT) Deakin University Geelong Victoria Australia; ^2^ Barwon Health Geelong Victoria Australia; ^3^ Florey Institute of Neuroscience and Mental Health Parkville Australia; ^4^ Orygen The National Centre of Excellence in Youth Mental Health, Centre for Youth Mental Health Parkville Australia; ^5^ Institute of Clinical Medicine, Psychiatry University of Eastern Finland Kuopio Finland; ^6^ Institute of Clinical Medicine, Kuopio Musculoskeletal Research Unit (KMRU) University of Eastern Finland Kuopio Finland; ^7^ Department of Medicine‐Western Health The University of Melbourne St Albans Victoria Australia; ^8^ School of Public Health and Preventative Medicine Monash University Melbourne Victoria Australia

**Keywords:** bipolar disorder, mental health, muscle strength, psychiatry, sarcopenia

## Abstract

**Introduction:**

While bipolar disorder is associated with significant comorbidity, little is known about the musculoskeletal health of individuals with bipolar disorder. Thus, we aimed to investigate the association between bipolar disorder and muscle strength, mass, quality and sarcopenia.

**Methods:**

Women with bipolar disorder (*n* = 106) were recruited from healthcare settings and age‐matched to controls (*n* = 300), without bipolar disorder, from the Geelong Osteoporosis Study. Both groups, aged ≥ 20 years. were drawn from South‐Eastern Australia. Bipolar disorder was identified using a clinical interview (SCID‐I/NP). Maximum handgrip strength (HGS) was measured using a Jamar dynamometer, appendicular lean mass (ALM) using a Lunar densitometer and body mass index (BMI, kg/m^2^) was calculated. Foundation for the National Institute of Health (FNIH) and European Working Group on Sarcopenia in Older People (EWGSOP2) algorithms were used to determine low HGS, ALM, probable and confirmed sarcopenia. Socioeconomic status (SES) was determined and information on lifestyle obtained via questionnaire.

**Results:**

Higher proportion of women with bipolar disorder met criteria for low HGS and ALM compared to those without bipolar disorder. Using FNIH and EWGSOP2 criteria, 36 (8.9%; 34 probable and 2 confirmed) and 46 (11.4%; 43 probable and 3 confirmed) women met criteria for sarcopenia, respectively. Women with bipolar disorder were twice as likely to have sarcopenia (FNIH; OR 2.24, 95% CI: 1.11–4.55 and EWGSOP2; OR 2.78, 95% CI: 1.47–5.20), independent of age, smoking, activity, alcohol, diet and SES.

**Conclusion:**

These data suggest women with bipolar disorder have an increased likelihood of sarcopenia. Replication and research into underlying mechanisms are next necessary steps.

## 1 Introduction

Bipolar disorder is associated with significant physical and mental comorbidity. This in turn has been shown to be associated with excess mortality and reduced life expectancy by up to 12 years [1]. It is increasingly understood that the risk factors and operative biological pathways for mental disorders overlap with those for common physical comorbidities [2]. While bipolar disorder has been identified as a risk factor for several physical conditions, in particular cardiovascular and metabolic disorders [3], little is known about the musculoskeletal health of individuals with bipolar disorder.

Recently, the authors and others have found those with bipolar disorder to have reduced bone mineral density (BMD) [4] and increased fracture risk [5] compared to those without. Whether those with bipolar disorder are at risk of other musculoskeletal conditions, such as sarcopenia, is relatively unknown. Sarcopenia is an age‐related, progressive condition characterised by the reduction of skeletal muscle strength, mass and physical function [6]. It is associated with greater disability and a wide range of adverse consequences, such as falls, hospitalisation, morbidity and mortality [7]. According to a recent meta‐analysis including 151 studies, up to 27% of those aged ≥ 60 years are considered sarcopenic, with this number expected to rise dramatically with the ageing population [8]. Despite its significance, a consensus for diagnostic criteria for sarcopenia is yet to be established. Among those available, the European working group on sarcopenia in older people (EWGSOP1 and EWGSOP2) [9, 10] and the Foundation for the National Institutes of Health (FNIH) [11] are arguably the most recognised and incorporate measures of muscle strength and mass.

Thus, in a group of population‐based women, we aimed to investigate the association between bipolar disorder, indices of muscle strength, mass, and quality and sarcopenia according to the Foundation for the National Institutes of Health (FNIH) and European working group on sarcopenia in older people (EWGSOP2) algorithms.

## 2 Methods

### 2.1 Participants

Women with bipolar disorder (*n* = 106) were recruited from public and/or private healthcare settings and age‐matched to controls (*n* = 300), without bipolar disorder, from the Geelong Osteoporosis Study, a population‐based cohort study. Both groups, aged 20 years and over, were drawn from the Barwon Statistical Division located in South‐Eastern Australia. Further details regarding recruitment and methodology are published elsewhere [12]. The study was approved by Barwon Health's Human Research Ethics Committee and written informed consent was obtained from all participants.

### 2.2 Measures

#### 2.2.1 Dependant Variables

*Muscle strength*: Maximum handgrip strength (HGS; kg) was measured using a handheld dynamometer (Jamar, Sammons Preston, Bolingbrook, IL). Participants were seated and asked to hold the dynamometer with their arm flexed at the elbow to 90 degrees. Participants were instructed to squeeze the device as hard as possible with the peak reading being recorded in duplicate. This procedure was repeated using the other hand, with the maximum reading used for this analysis.

*Lean mass*: Appendicular lean mass (ALM; sum of lean mass of the arms and legs) was obtained from whole body dual‐energy x‐ray absorptiometry (DXA; Lunar Prodigy‐Pro, Madison, WI, USA) scans. ALM (kg) was expressed relative to height (ALM/height; kg/m^2^) and body mass index (ALM/BMI; kg/m^2^).

*Muscle quality*: Muscle Quality Index (MQI) was calculated by dividing HGS (kg) by ALM (kg) to estimate muscle strength per unit of muscle mass [13].

*Sarcopenia*: Sarcopenia was defined according to the Foundation for the National Institutes of Health (FNIH) [11] and European Working Group on Sarcopenia in Older People (EWGSOP2) [10] algorithms. Based on FNIH, low HGS was considered as < 16 kg and low muscle mass as < 0.512m^2^ (ALM adjusted for BMI) and based on EWGSOP2, low HGS was considered as < 16 kg and low lean muscle mass as < 15 kg/m^2^ (ALM adjusted for height). Probable sarcopenia was determined by low HGS and confirmed sarcopenia by the presence of both low HGS and low muscle mass for each definition.

#### 2.2.2 Independent Variables

The Structured Clinical Interview for Diagnostic and Statistical Manual of Mental Disorders, Fourth Edition, Non‐patient edition (SCID‐I/NP) was used to identify a lifetime history of bipolar disorder [14]. Trained personnel with postgraduate qualifications in psychology conducted the interviews under the supervision of a clinically trained psychologist.

Weight and height were measured to the nearest 0.1 kg and 0.01 m using electronic scales and a wall‐mounted Harpenden stadiometer, respectively. BMI was calculated as body mass/height^2^ (kg/m^2^).

Area based socioeconomic status (SES) was determined using the Index of Relative Socio‐economic Advantage and Disadvantage (IRSAD) derived from Socio‐Economic Index for Areas (SEFA) scores from the Australian Bureau of Statistics census data. The IRSAD identifies those of greater disadvantage (Quintile 1) through to greater advantage (Quintile 5) [15].

Tobacco smoking status (never, past, current) was self‐reported at the time of appointment.

Physical activity was assessed using a 7‐point scale ranging from ‘very active’ (moves, walks and works energetically and participates in vigorous activity) through to ‘bed fast’ (unable to walk); for these analyses participants were categorised as ‘active’ if vigorous or light exercise was performed regularly or otherwise ‘sedentary’ [16].

The Dietary Questionnaire for Epidemiological Studies (DQES) developed by the Cancer Council Victoria was used to assess dietary intake and alcohol consumption (g/day) [17]. The DQES is a validated food frequency questionnaire that assesses ‘usual’ diet over a 12 months period. From these data, the Australian Recommended Food Score (ARFS) was calculated, with scores ranging between 0 and 74 reflecting alignment with the Australian Dietary Guidelines. A higher score indicates better diet quality [18].

### 2.3 Statistical Analyses

Differences between those with and without bipolar disorder were determined using *t*‐tests for parametric variables, Kruskal–Wallis for non‐parametric continuous variables and chi‐square tests or Fisher's exact test for discrete variables. Multiple logistic regression models were used to determine the associations between bipolar disorder and sarcopenia while testing for potential confounding. Age, SES, smoking, physical activity, alcohol consumption and diet quality were tested in the models for confounding or effect modification. Statistical analyses were performed using Minitab (version 18, Minitab, State College, PA, USA).

## 3 Results

Characteristics of the whole group and those with and without bipolar disorder are shown in Table 1. Participants were aged between 21 and 80 years, with a median age of 50 years. There were differences between those with and without bipolar disorder in regard to weight, smoking status, diet quality, HGS and muscle quality (MQI); otherwise, the groups were similar.

**TABLE 1:** Characteristics of all participants and those with and without bipolar disorder.

		Bipolar disorder		
	All	Yes	No	*p*
	*n* = 406	*n* = 106	*n* = 300	
Age (yr)	49.8 (40.7–57.4)	48.2 (41.5–57.2)	50.0 (39.3–57.5)	0.273
Weight (kg)	72.4 (63.5–86.2)	75.3 (65.4–89.9)	71.6 (62.3–85.0)	0.031
Height (m)	1.63 ± 0.06	1.64 ± 0.06	1.63 ± 0.06	0.196
BMI (kg/m^2^)	27.2 (23.7–32.0)	28.0 (24.1–34.0)	26.9 (23.7–31.4)	0.108
Mobility (active)	317 (79.3%)	78 (73.6%)	239 (81.3%)	0.093
Smoking				< 0.001
Never	200 (49.6%)	38 (33.3%)	162 (54.6%)	
Past	131 (32.5%)	36 (34.0%)	95 (32.0%)	
Current	72 (17.9%)	32 (30.2%)	40 (13.5%)	
Alcohol intake (g/d)	3.7 (0.5–14.0)	3.8 (0.5–14.3)	3.7 (0.5–14.1)	0.655
Diet quality	33.0 (26.0–39.0)	28.0 (23.0–34.0)	34.0 (28.0–40.0)	< 0.001
Socioeconomic status				0.079
Quintile 1 (lowest)	60 (14.9%)	15 (14.4%)	45 (15.0%)	
Quintile 2	41 (10.2%)	18 (17.3%)	23 (7.7%)	
Quintile 3	151 (37.4%)	37 (35.6%)	114 (38.0%)	
Quintile 4	97 (24.0%)	23 (22.1%)	74 (24.7%)	
Quintile 5	55 (13.6%)	11 (10.6%)	44 (14.7%)	
Muscle indices				
Handgrip strength (kg)	27.4 ± 6.4	25.3 ± 6.1	28.2 ± 6.4	< 0.001
ALM (kg)	18.3 ± 2.6	18.6 ± 2.7	18.2 ± 2.6	0.275
MQI (kg)	1.5 ± 0.3	1.4 ± 0.3	1.6 ± 0.3	< 0.001

*Note:* Values are given as median (interquartile range), mean ± standard deviation or *n* (%).

A higher proportion of women with bipolar disorder met criteria for low HGS [FNIH and EWGSOP2; 15 (14.2%) vs. 21 (7.1%), *p* = 0.027], low muscle mass [FNIH; 13 (12.3%) vs. 18 (6.0%), *p* = 0.037 and EWGSOP2; 6 (5.7%) vs. 7 (2.3%), *p* = 0.09] and had a lower MQI [1.37 ± 0.34 vs. 1.56 ± 0.32, *p* ≤ 0.001] compared to those without bipolar disorder.

Using FNIH and EWGSOP2 criteria, 36 women (8.9%; 34 probable and 2 confirmed) and 46 women (11.4%; 43 probable and 3 confirmed) met criteria for sarcopenia, respectively. Compared to women without bipolar disorder, women with bipolar disorder were twice as likely to have sarcopenia irrespective of criteria used (FNIH; OR 2.24, 95% CI: 1.11–4.55, *p* = 0.025 and EWGSOP2; OR 2.78, 95% CI: 1.47–5.20, *p* = 0.002). These relationships were independent of age, SES, smoking, physical activity, alcohol consumption and diet quality. No effect modification was identified.

## 4 Discussion

Bipolar disorder was associated with poorer musculoskeletal health. A higher proportion of women with bipolar disorder met criteria for low handgrip strength and low lean mass, had poorer muscle quality and were twice as likely to have sarcopenia compared to those without bipolar disorder. These relationships were independent of age, SES, smoking, physical activity, alcohol consumption and diet quality.

To date, only two studies have investigated the association between bipolar disorder and sarcopenia or its components. In a sample of 111 men and women aged 18–68 years with bipolar disorder consecutively recruited from a psychiatry outpatients' clinic at Çukurova University in Turkey, according to EWGSOP criteria, sarcopenia was present, with the prevalence of pre sarcopenia, sarcopenia and severe sarcopenia being 6.3%, 9.0% and 1.8%, respectively [7]. Interestingly, those with sarcopenia were more likely to have a greater number of episodes than those without sarcopenia, indicating severity may play a role. Although given a comparison group was not included, whether the reported figures are indeed higher than the general population cannot be extrapolated. In further support, a case control study of 271,118 adults (mean age 56 years; 50% female) participating in the UK biobank found handgrip strength to be lower for men and women with bipolar disorder compared to healthy controls [19].

Whilst evidence concerning the association between bipolar disorder and sarcopenia is in its infancy, others have investigated whether unipolar depression and/or depressive symptoms are associated with sarcopenia and/or its components. A recently conducted meta‐analysis including 11 studies revealed that depression, among other diseases, sociodemographic and behavioural factors, was an independent risk factor for sarcopenia in older community‐dwelling adults (OR = 1.46, 95% CI: 1.17–1.83) [20]. Furthermore, in a previous study, we found women, but not men, with current depressive symptoms as measured using the Hospital Anxiety and Depression Scale had lower HGS according to criteria of the EWGSOP2, FNIH and Sarcopenia Definitions and Outcomes Consortium (SDOC) compared to those without depressive symptoms [21]. Evidence for sarcopenia giving rise to depressive disorders and symptoms also exists. After adjusting for potential confounders including age, sex, cognitive performance and physical activity, sarcopenia was associated with an increased risk of depression in a meta‐analysis including six studies (adjusted OR: 1.82, 95% CI: 1.16–2.86) [22]. Similarly, grip strength and ALM have been independently associated with an increased risk of depressive symptoms in a group of 4871 adults participating in the National Health and Nutrition Examination Survey [23]. An emerging marker of health and physical function, muscle quality index (MQI), was also found to be associated with an independent reduced risk of depression among adults aged < 60 years residing in the US [24].

Evidence is accumulating suggesting a causal, bidirectional relationship between depression and sarcopenia‐related traits. A mendelian randomisation (MR) analysis was recently conducted finding a negative association between depression and ALM (OR 0.93, 95% CI 0.89–0.98, *p* = 0.005) and handgrip strength to be associated with depression (OR 0.20, 95% CI 0.11–0.37, *p* < 0.001), concluding depression is causally related to decreased muscle mass and lower muscle strength leads to a greater risk of depression [25]. Other MR analyses conducted the same year found grip strength and usual walking speed to have a direct causal effect on MDD [26], while MDD was found to have a direct causal effect on ALM [26, 27].

Potential explanations for the observed relationship between bipolar disorder and sarcopenia likely involve shared genetic and other biological pathways and lifestyle factors. A meta‐analysis of 52 studies found brain‐derived neurotrophic factor (BDNF), a protein associated with hippocampal neurogenesis and expressed in skeletal muscle, to be associated with disease activity in bipolar disorder [28]. Furthermore, chronic low‐grade inflammation similarly has been shown to be a core feature of bipolar disorder as with a number of physical conditions including sarcopenia [29, 30]. Age‐related muscle wasting is known to be associated with mitochondrial dysfunction [31] which has been proposed as a potential explanatory mechanism associated with manic episodes in bipolar disorder [32]. Lastly, observed differences could also be due to lifestyle factors such as physical inactivity, smoking, increased alcohol consumption, and poor diet, in turn, predisposing those with bipolar disorder to poorer muscle health.

A strength of the study is that those with and without bipolar disorder were drawn from the same underlying population. The objective measures of muscle strength and mass and a gold standard clinical interview to determine bipolar disorder are further strengths. However, limitations need to be taken into consideration when interpreting the study findings. The cross‐sectional study design precludes determining whether a causal relationship exists. Furthermore, findings may not be generalisable to other populations given our sample included women only and predominantly white Australian women living in the community. While we attempted to account for confounding in our analysis, there may still be unknown factors at play, and we were not able to examine blood biomarkers to test the mechanistic hypothesis raised above. In addition, differential recall bias is possible due to self‐report of some lifestyle factors. Lastly, as with all observation studies, a ‘healthy’ bias may need to be considered; therefore, it is possible that the associations observed could be underestimated.

In conclusion, women with bipolar disorder were more likely to meet criteria for low handgrip strength and muscle mass and were twice as likely to have sarcopenia compared to those without bipolar disorder. Following replication, longitudinal research into underlying mechanisms and clinical studies of physical activity and resistance training, development of clinical practice guidelines including exercise and dietary interventions could be the next necessary steps.

## Author Contributions

Conception and design: Lana J. Williams, Amanda L. Stuart, Julie A. Pasco. Development of the methodology: Lana J. Williams, Amanda L. Stuart, Julie A. Pasco. Analyses: Lana J. Williams. Draft and review: Lana J. Williams, Amanda L. Stuart, Michael Berk, Shae E. Quirk, Emma C. West, Heli Koivumaa‐Honkanen, Risto Honkanen, Jeremi Heikkinen, Jessica A. Davis, Kavindi Weerasinghe, Julie A. Pasco. All authors read and approved the final version.

## Funding

This study is supported by a competitive project grant from the National Health and Medical Research Council (NHMRC; 1104438). LJW is supported by an NHMRC Emerging Leadership 2 Investigator grant (1174060) and MB is supported by a NHMRC Leadership 3 Investigator grant (2017131). HKH and SQ are supported by the Signe and Ane Gyllenberg's Foundation (5799) and Päivikki and Sakari Sohlberg's Foundation (20035). HKH is also supported by Ane Gyllenberg's Foundation (6671).

## Ethics Statement

The study was approved by Barwon Health's Human Research Ethics Committee (project 10/89) and written informed consent was obtained from all participants.

## Conflicts of Interest

The authors declare no conflicts of interest.

## Data Availability

The data that support the findings of this study are available from the corresponding author upon reasonable request.
